# [*N*,*N*′-Bis(2,6-diethyl-4-phenyl­phen­yl)butane-2,3-di­imine-κ^2^ *N*,*N*′]di­bromido­nickel(II)

**DOI:** 10.1107/S160053681400292X

**Published:** 2014-02-15

**Authors:** Jianchao Yuan, Jingjing Xia, Weibing Xu, Yanqiong Mu

**Affiliations:** a Key Laboratory of Eco-Environment-Related Polymer Materials of Ministry of, Education, Key Laboratory of Polymer Materials of Gansu Province, College of Chemistry & Chemical Engineering, Northwest Normal University, Lanzhou 730070, People’s Republic of China

## Abstract

The complex molecule in the title compound, [NiBr_2_(C_36_H_40_N_2_)], has mirror symmetry. The Ni^II^ atom and two Br atoms are located on the mirror plane. The Ni^II^ atom is four-coordinated by the two Br atoms and two N atoms from an *N*,*N*′-bis(2,6-diethyl-4-phenyl­phen­yl)butane-2,3-di­imine ligand in a distorted tetra­hedral geometry. The dihedral angle formed between the two adjacent benzene rings is 47.1 (1)°.

## Related literature   

For background to α-di­imine nickel catalysts, see: Johnson *et al.* (1995 ▶); Killian *et al.* (1996 ▶). For the effect of ligand structure on the reactivity of organometallic complexes, see: Popeney & Guan (2010 ▶); Popeney *et al.* (2011 ▶).
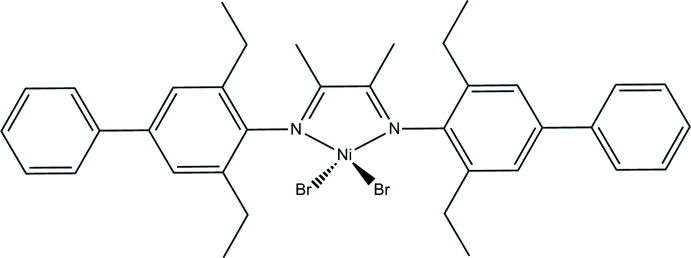


## Experimental   

### 

#### Crystal data  

[NiBr_2_(C_36_H_40_N_2_)]
*M* *_r_* = 719.19
Orthorhombic, 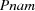

*a* = 15.6587 (5) Å
*b* = 6.9359 (3) Å
*c* = 30.1928 (16) Å
*V* = 3279.2 (2) Å^3^
*Z* = 4
Mo *K*α radiation
μ = 3.06 mm^−1^
*T* = 293 K
0.42 × 0.38 × 0.35 mm

#### Data collection  

Oxford Diffraction SuperNova CCD diffractometer
Absorption correction: multi-scan (*CrysAlis PRO*; Oxford Diffraction, 2012 ▶) *T*_min_ = 0.508, *T*_max_ = 1.000
10334 measured reflections
3415 independent reflections
2376 reflections with *I* > 2σ(*I*)
*R*_int_ = 0.046

#### Refinement  

*R*[*F* ^2^ > 2σ(*F* ^2^)] = 0.043
*wR*(*F* ^2^) = 0.096
*S* = 1.02
3415 reflections
193 parameters
H-atom parameters constrained
Δρ_max_ = 0.53 e Å^−3^
Δρ_min_ = −0.53 e Å^−3^

### 

Data collection: *CrysAlis PRO* (Oxford Diffraction, 2012 ▶); cell refinement: *CrysAlis PRO*; data reduction: *CrysAlis PRO*; program(s) used to solve structure: *SHELXS97* (Sheldrick, 2008 ▶); program(s) used to refine structure: *SHELXL97* (Sheldrick, 2008 ▶); molecular graphics: *SHELXTL* (Sheldrick, 2008 ▶); software used to prepare material for publication: *SHELXTL*.

## Supplementary Material

Crystal structure: contains datablock(s) I, New_Global_Publ_Block. DOI: 10.1107/S160053681400292X/hy2642sup1.cif

Structure factors: contains datablock(s) I. DOI: 10.1107/S160053681400292X/hy2642Isup2.hkl

CCDC reference:

Additional supporting information:  crystallographic information; 3D view; checkCIF report

## Figures and Tables

**Table 1:** Selected bond lengths (Å)

Ni1—N1	1.991 (2)
Ni1—Br2	2.3575 (9)
Ni1—Br3	2.3173 (8)

**Fig. 1.**
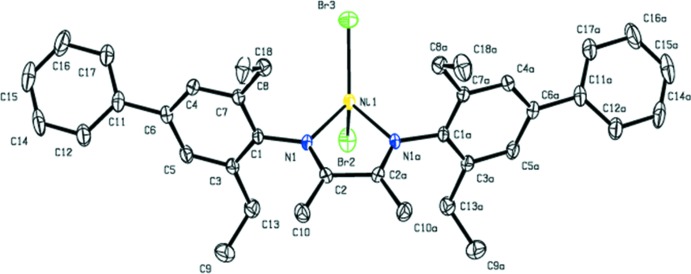
Molecular structure of the title compound, showing the 30% probability level ellipsoids. [Symmetry code: (a) x, y, 3/2-z.]

## supplementary crystallographic information

### 1. Comment

There is a considerable interest in the development of new late transition metal catalysts for the polymerization of α-olefins since Brookhart *et al.* discovered highly active α-diimine nickel catalysts (Johnson *et al.*, 1995; Killian *et al.*, 1996). The ligand structure has a dramatic effect on the reactivity of organometallic complexes (Popeney *et al.*, 2011; Popeney & Guan, 2010). Advances in the field of homogeneous catalysis have led to the synthesis of well defined transition metal complexes capable of catalyzing a wide range of organic transformations. It is well known that the Lewis acid catalyzed Friedel-Crafts alkylation of substituted aromatic rings is a highly versatile C—C bond forming method. In this study, we designed and synthesized the title compound, and its molecular structure was characterized by X-ray diffraction. The dihedral angle formed between the benzene ring and phenylethyl ring is 47.1 (1)°.

### 2. Experimental

Formic acid (0.5 ml) was added to a stirred solution of 2,3-butanedione (0.09 g, 1.00 mmol) and 2,6-diethyl-4-phenylbenzenamine (0.45 g, 2.00 mmol) in ethanol (10 ml). The mixture was refluxed for 24 h, then cooled and the precipitate was separated by filtration. The solid was recrystallized from EtOH/CH_2_Cl_2_ (v/v, 10:1), washed and dried under vacuum to give bis[*N*,*N*'-(2,6-diethyl-4-(1-phenyl)imino]-1,2-dimethylethane (yield: 0.69 g, 85%). Analysis, calculated for C_36_H_40_N_2_: C 86.35, H 8.05, N 5.59%; found: C 84.96, H 7.21, N 7.82%.

NiBr_2_(DME) (0.13 g, 1.20 mmol), bis[*N,N*'-(2,6-diethyl-4-(1-phenyl)imino]-1,2-dimethylethane (0.20 g, 4.00 mmol) and dichloromethane (40 ml) were mixed in a Schlenk flask and stirred at room temperature for 24 h. The resulting suspension was filtered. The solvent was removed under vacuum and the residue was washed with diethyl ether (15 ml) three times, and then dried under vacuum at room temperature to give the title compound (yield: 0.63 g, 82%). Analysis, calculated for C_36_H_40_Br_2_N_2_Ni: C 60.12, H 5.61, N 3.89%; found: C 59.88, H 5.31, N 3.56%. FT-IR (KBr, cm^-1^): 1649 (C═N). Crystals suitable for X-ray structure determination were grown from a solution of the title compound in a mixture of cyclohexane/dichloromethane (v/v, 1:4).

### 3. Refinement

H atoms were placed in calculated positions and refined as riding atoms, with C—H = 0.93 (aromatic), 0.97 (CH_2_) and 0.96 (CH_3_) Å and with *U*_iso_(H) = 1.2(1.5 for methyl)*U*_eq_(C).

### Crystal data

**Table:**

[NiBr_2_(C_36_H_40_N_2_)]	*D*_x_ = 1.457 Mg m^−^^3^
*M**_r_* = 719.19	Mo *K*α radiation, λ = 0.71073 Å
Orthorhombic, *P**n**a**m*	Cell parameters from 2436 reflections
*a* = 15.6587 (5) Å	θ = 3.5–26.1°
*b* = 6.9359 (3) Å	µ = 3.06 mm^−^^1^
*c* = 30.1928 (16) Å	*T* = 293 K
*V* = 3279.2 (2) Å^3^	Block, brown
*Z* = 4	0.42 × 0.38 × 0.35 mm
*F*(000) = 1472	

### Data collection

**Table:**

Oxford Diffraction SuperNova CCD diffractometer	3415 independent reflections
Radiation source: fine-focus sealed tube	2376 reflections with *I* > 2σ(*I*)
Graphite monochromator	*R*_int_ = 0.046
ω scans	θ_max_ = 26.4°, θ_min_ = 3.0°
Absorption correction: multi-scan (*CrysAlis PRO*; Oxford Diffraction, 2012)	*h* = −19→18
*T*_min_ = 0.508, *T*_max_ = 1.000	*k* = −8→4
10334 measured reflections	*l* = −37→22

### Refinement

**Table:**

Refinement on *F*^2^	Primary atom site location: structure-invariant direct methods
Least-squares matrix: full	Secondary atom site location: difference Fourier map
*R*[*F*^2^ > 2σ(*F*^2^)] = 0.043	Hydrogen site location: inferred from neighbouring sites
*wR*(*F*^2^) = 0.096	H-atom parameters constrained
*S* = 1.02	*w* = 1/[σ^2^(*F*_o_^2^) + (0.0361*P*)^2^ + 2.0948*P*] where *P* = (*F*_o_^2^ + 2*F*_c_^2^)/3
3415 reflections	(Δ/σ)_max_ < 0.001
193 parameters	Δρ_max_ = 0.53 e Å^−^^3^
0 restraints	Δρ_min_ = −0.53 e Å^−^^3^

### Special details

**Table:**

Geometry. All e.s.d.'s (except the e.s.d. in the dihedral angle between two l.s. planes) are estimated using the full covariance matrix. The cell e.s.d.'s are taken into account individually in the estimation of e.s.d.'s in distances, angles and torsion angles; correlations between e.s.d.'s in cell parameters are only used when they are defined by crystal symmetry. An approximate (isotropic) treatment of cell e.s.d.'s is used for estimating e.s.d.'s involving l.s. planes.
Refinement. Refinement of *F*^2^ against ALL reflections. The weighted *R*-factor *wR* and goodness of fit *S* are based on *F*^2^, conventional *R*-factors *R* are based on *F*, with *F* set to zero for negative *F*^2^. The threshold expression of *F*^2^ > σ(*F*^2^) is used only for calculating *R*-factors(gt) *etc*. and is not relevant to the choice of reflections for refinement. *R*-factors based on *F*^2^ are statistically about twice as large as those based on *F*, and *R*- factors based on ALL data will be even larger.

### Fractional atomic coordinates and isotropic or equivalent isotropic displacement parameters (Å^2^)

**Table:**

	*x*	*y*	*z*	*U*_iso_*/*U*_eq_	
Ni1	0.34408 (3)	0.82145 (10)	0.7500	0.03485 (18)	
Br2	0.28258 (4)	0.51120 (8)	0.7500	0.05577 (19)	
Br3	0.49179 (3)	0.84183 (12)	0.7500	0.0705 (2)	
N1	0.26850 (15)	0.9588 (4)	0.70741 (8)	0.0293 (6)	
C1	0.28083 (18)	0.9383 (5)	0.66015 (10)	0.0292 (7)	
C2	0.20665 (19)	1.0521 (5)	0.72490 (10)	0.0311 (8)	
C3	0.23084 (19)	0.8070 (5)	0.63611 (11)	0.0330 (8)	
C4	0.3703 (2)	0.9915 (5)	0.59746 (11)	0.0345 (8)	
H4	0.4167	1.0528	0.5844	0.041*	
C5	0.2544 (2)	0.7728 (5)	0.59207 (11)	0.0379 (8)	
H5	0.2217	0.6882	0.5753	0.046*	
C6	0.3243 (2)	0.8598 (5)	0.57262 (11)	0.0347 (8)	
C7	0.34987 (18)	1.0362 (5)	0.64119 (10)	0.0309 (7)	
C8	0.3998 (2)	1.1906 (5)	0.66489 (12)	0.0420 (9)	
H8A	0.3808	1.1971	0.6954	0.050*	
H8B	0.4598	1.1556	0.6650	0.050*	
C9	0.0769 (2)	0.7102 (8)	0.62840 (16)	0.0748 (15)	
H9A	0.0857	0.6469	0.6005	0.112*	
H9B	0.0627	0.8430	0.6234	0.112*	
H9C	0.0310	0.6482	0.6440	0.112*	
C10	0.1368 (2)	1.1541 (6)	0.70058 (12)	0.0476 (9)	
H10A	0.0831	1.0933	0.7070	0.071*	
H10B	0.1475	1.1479	0.6693	0.071*	
H10C	0.1348	1.2865	0.7098	0.071*	
C11	0.3499 (2)	0.8120 (5)	0.52645 (11)	0.0411 (9)	
C12	0.2902 (3)	0.8027 (6)	0.49250 (12)	0.0540 (11)	
H12	0.2330	0.8278	0.4984	0.065*	
C13	0.1566 (2)	0.6983 (6)	0.65535 (12)	0.0475 (10)	
H13A	0.1725	0.5639	0.6585	0.057*	
H13B	0.1448	0.7483	0.6847	0.057*	
C14	0.3154 (3)	0.7564 (7)	0.44983 (13)	0.0664 (13)	
H14	0.2752	0.7525	0.4272	0.080*	
C15	0.3988 (4)	0.7166 (7)	0.44081 (14)	0.0742 (15)	
H15	0.4151	0.6847	0.4121	0.089*	
C16	0.4587 (3)	0.7232 (8)	0.47362 (15)	0.0754 (15)	
H16	0.5156	0.6956	0.4673	0.091*	
C17	0.4343 (3)	0.7713 (7)	0.51643 (13)	0.0603 (12)	
H17	0.4753	0.7763	0.5387	0.072*	
C18	0.3901 (3)	1.3857 (6)	0.64409 (15)	0.0692 (13)	
H18A	0.4087	1.3804	0.6138	0.104*	
H18B	0.4241	1.4776	0.6600	0.104*	
H18C	0.3312	1.4240	0.6451	0.104*	

### Atomic displacement parameters (Å^2^)

**Table:**

	*U*^11^	*U*^22^	*U*^33^	*U*^12^	*U*^13^	*U*^23^
Ni1	0.0328 (3)	0.0534 (4)	0.0183 (3)	0.0117 (3)	0.000	0.000
Br2	0.0802 (4)	0.0450 (3)	0.0421 (3)	0.0080 (3)	0.000	0.000
Br3	0.0341 (3)	0.1250 (6)	0.0523 (4)	0.0136 (3)	0.000	0.000
N1	0.0306 (13)	0.0418 (16)	0.0154 (12)	0.0013 (12)	0.0013 (11)	−0.0002 (12)
C1	0.0321 (16)	0.0389 (19)	0.0165 (15)	0.0037 (15)	−0.0012 (13)	0.0011 (14)
C2	0.0314 (16)	0.0382 (19)	0.0236 (17)	−0.0002 (14)	−0.0044 (14)	0.0020 (15)
C3	0.0342 (16)	0.041 (2)	0.0241 (17)	−0.0025 (15)	−0.0030 (14)	0.0060 (16)
C4	0.0348 (16)	0.045 (2)	0.0239 (16)	−0.0035 (16)	0.0037 (15)	0.0028 (17)
C5	0.0451 (19)	0.044 (2)	0.0244 (17)	−0.0068 (17)	−0.0058 (16)	−0.0037 (17)
C6	0.0417 (18)	0.041 (2)	0.0209 (16)	0.0002 (16)	−0.0033 (15)	−0.0004 (16)
C7	0.0334 (16)	0.037 (2)	0.0222 (16)	0.0030 (15)	−0.0022 (14)	−0.0015 (15)
C8	0.0433 (19)	0.049 (2)	0.0339 (19)	−0.0061 (18)	−0.0022 (16)	−0.0062 (19)
C9	0.050 (2)	0.118 (4)	0.056 (3)	−0.031 (3)	−0.008 (2)	0.008 (3)
C10	0.048 (2)	0.061 (2)	0.0336 (19)	0.0162 (19)	−0.0063 (17)	0.003 (2)
C11	0.060 (2)	0.042 (2)	0.0215 (17)	−0.0061 (18)	0.0019 (17)	−0.0008 (17)
C12	0.068 (2)	0.066 (3)	0.028 (2)	−0.016 (2)	−0.0033 (19)	−0.002 (2)
C13	0.049 (2)	0.060 (3)	0.034 (2)	−0.014 (2)	0.0001 (17)	0.009 (2)
C14	0.102 (4)	0.076 (3)	0.022 (2)	−0.026 (3)	−0.013 (2)	−0.003 (2)
C15	0.124 (4)	0.072 (3)	0.027 (2)	−0.011 (3)	0.019 (3)	−0.007 (2)
C16	0.090 (3)	0.098 (4)	0.038 (3)	0.010 (3)	0.024 (3)	−0.007 (3)
C17	0.063 (3)	0.090 (3)	0.028 (2)	0.009 (2)	0.0031 (19)	−0.007 (2)
C18	0.104 (3)	0.053 (3)	0.050 (3)	−0.022 (3)	0.011 (3)	−0.009 (2)

### Geometric parameters (Å, º)

**Table:**

Ni1—N1	1.991 (2)	C9—H9A	0.9600
Ni1—Br2	2.3575 (9)	C9—H9B	0.9600
Ni1—Br3	2.3173 (8)	C9—H9C	0.9600
N1—C2	1.279 (4)	C10—H10A	0.9600
N1—C1	1.447 (4)	C10—H10B	0.9600
C1—C7	1.399 (4)	C10—H10C	0.9600
C1—C3	1.403 (4)	C11—C17	1.386 (5)
C2—C10	1.496 (4)	C11—C12	1.388 (5)
C2—C2^i^	1.516 (6)	C12—C14	1.385 (6)
C3—C5	1.400 (5)	C12—H12	0.9300
C3—C13	1.503 (4)	C13—H13A	0.9700
C4—C6	1.384 (5)	C13—H13B	0.9700
C4—C7	1.393 (4)	C14—C15	1.363 (6)
C4—H4	0.9300	C14—H14	0.9300
C5—C6	1.381 (5)	C15—C16	1.365 (7)
C5—H5	0.9300	C15—H15	0.9300
C6—C11	1.488 (4)	C16—C17	1.389 (5)
C7—C8	1.507 (4)	C16—H16	0.9300
C8—C18	1.499 (6)	C17—H17	0.9300
C8—H8A	0.9700	C18—H18A	0.9600
C8—H8B	0.9700	C18—H18B	0.9600
C9—C13	1.492 (5)	C18—H18C	0.9600
			
N1^i^—Ni1—N1	80.49 (14)	C13—C9—H9C	109.5
N1^i^—Ni1—Br3	124.35 (7)	H9A—C9—H9C	109.5
N1—Ni1—Br3	124.35 (7)	H9B—C9—H9C	109.5
N1^i^—Ni1—Br2	101.19 (8)	C2—C10—H10A	109.5
N1—Ni1—Br2	101.19 (8)	C2—C10—H10B	109.5
Br3—Ni1—Br2	117.61 (4)	H10A—C10—H10B	109.5
C2—N1—C1	123.9 (3)	C2—C10—H10C	109.5
C2—N1—Ni1	115.2 (2)	H10A—C10—H10C	109.5
C1—N1—Ni1	120.70 (19)	H10B—C10—H10C	109.5
C7—C1—C3	122.3 (3)	C17—C11—C12	118.1 (3)
C7—C1—N1	117.3 (3)	C17—C11—C6	120.4 (3)
C3—C1—N1	120.0 (3)	C12—C11—C6	121.4 (3)
N1—C2—C10	126.2 (3)	C14—C12—C11	120.4 (4)
N1—C2—C2^i^	114.40 (17)	C14—C12—H12	119.8
C10—C2—C2^i^	119.41 (18)	C11—C12—H12	119.8
C5—C3—C1	117.0 (3)	C9—C13—C3	114.1 (3)
C5—C3—C13	119.1 (3)	C9—C13—H13A	108.7
C1—C3—C13	123.9 (3)	C3—C13—H13A	108.7
C6—C4—C7	122.7 (3)	C9—C13—H13B	108.7
C6—C4—H4	118.6	C3—C13—H13B	108.7
C7—C4—H4	118.6	H13A—C13—H13B	107.6
C6—C5—C3	122.6 (3)	C15—C14—C12	120.4 (4)
C6—C5—H5	118.7	C15—C14—H14	119.8
C3—C5—H5	118.7	C12—C14—H14	119.8
C5—C6—C4	118.1 (3)	C14—C15—C16	120.5 (4)
C5—C6—C11	121.0 (3)	C14—C15—H15	119.8
C4—C6—C11	121.0 (3)	C16—C15—H15	119.8
C4—C7—C1	117.2 (3)	C15—C16—C17	119.6 (4)
C4—C7—C8	119.3 (3)	C15—C16—H16	120.2
C1—C7—C8	123.5 (3)	C17—C16—H16	120.2
C18—C8—C7	113.0 (3)	C11—C17—C16	121.0 (4)
C18—C8—H8A	109.0	C11—C17—H17	119.5
C7—C8—H8A	109.0	C16—C17—H17	119.5
C18—C8—H8B	109.0	C8—C18—H18A	109.5
C7—C8—H8B	109.0	C8—C18—H18B	109.5
H8A—C8—H8B	107.8	H18A—C18—H18B	109.5
C13—C9—H9A	109.5	C8—C18—H18C	109.5
C13—C9—H9B	109.5	H18A—C18—H18C	109.5
H9A—C9—H9B	109.5	H18B—C18—H18C	109.5
			
N1^i^—Ni1—N1—C2	−5.1 (3)	C7—C4—C6—C11	−178.2 (3)
Br3—Ni1—N1—C2	−130.5 (2)	C6—C4—C7—C1	1.4 (5)
Br2—Ni1—N1—C2	94.5 (2)	C6—C4—C7—C8	−176.1 (3)
N1^i^—Ni1—N1—C1	179.54 (19)	C3—C1—C7—C4	−3.0 (5)
Br3—Ni1—N1—C1	54.2 (3)	N1—C1—C7—C4	169.6 (3)
Br2—Ni1—N1—C1	−80.8 (2)	C3—C1—C7—C8	174.4 (3)
C2—N1—C1—C7	110.0 (4)	N1—C1—C7—C8	−13.0 (5)
Ni1—N1—C1—C7	−75.1 (3)	C4—C7—C8—C18	63.1 (4)
C2—N1—C1—C3	−77.2 (4)	C1—C7—C8—C18	−114.3 (4)
Ni1—N1—C1—C3	97.7 (3)	C5—C6—C11—C17	−132.5 (4)
C1—N1—C2—C10	0.4 (5)	C4—C6—C11—C17	46.9 (5)
Ni1—N1—C2—C10	−174.8 (3)	C5—C6—C11—C12	46.1 (5)
C1—N1—C2—C2^i^	179.5 (2)	C4—C6—C11—C12	−134.4 (4)
Ni1—N1—C2—C2^i^	4.3 (2)	C17—C11—C12—C14	−0.8 (6)
C7—C1—C3—C5	1.8 (5)	C6—C11—C12—C14	−179.5 (4)
N1—C1—C3—C5	−170.6 (3)	C5—C3—C13—C9	−52.4 (5)
C7—C1—C3—C13	179.7 (3)	C1—C3—C13—C9	129.8 (4)
N1—C1—C3—C13	7.2 (5)	C11—C12—C14—C15	1.0 (7)
C1—C3—C5—C6	1.1 (5)	C12—C14—C15—C16	−0.5 (7)
C13—C3—C5—C6	−176.8 (3)	C14—C15—C16—C17	−0.1 (8)
C3—C5—C6—C4	−2.7 (5)	C12—C11—C17—C16	0.2 (7)
C3—C5—C6—C11	176.8 (3)	C6—C11—C17—C16	178.9 (4)
C7—C4—C6—C5	1.3 (5)	C15—C16—C17—C11	0.3 (8)

Symmetry code: (i) *x*, *y*, −*z*+3/2.

## References

[bb1] Johnson, L. K., Killian, C. M. & Brookhart, M. (1995). *J. Am. Chem. Soc.* **117**, 6414–6415.

[bb2] Killian, C. M., Tempel, D. J., Johnson, L. K. & Brookhart, M. (1996). *J. Am. Chem. Soc.* **118**, 11664–11665.

[bb3] Oxford Diffraction (2012). *CrysAlis PRO* Agilent Technologies, Yarnton, England.

[bb4] Popeney, C. S. & Guan, Z. B. (2010). *Macromolecules*, **43**, 4091–4097.

[bb5] Popeney, C. S., Levins, C. M. & Guan, Z. B. (2011). *Organometallics*, **30**, 2432–2452.

[bb6] Sheldrick, G. M. (2008). *Acta Cryst.* A**64**, 112–122. 10.1107/S0108767307043930 18156677

